# Beyond Chordoma: A Comprehensive Review of Sacral Lesions

**DOI:** 10.3390/curroncol33020115

**Published:** 2026-02-15

**Authors:** Leonor Garbin Savarese, Nicolas Papalexis, Mateus de Andrade Hernandes, Giancarlo Facchini, Marco Miceli, Marcello Henrique Nogueira-Barbosa

**Affiliations:** 1 Department of Medical Imaging, Hematology and Clinical Oncology, Ribeirao Preto Medical School, University of Sao Paulo, Ribeirão Preto 14049-090, SP, Brazil; mateusah@alumni.usp.br (M.d.A.H.); marcello@fmrp.usp.br (M.H.N.-B.); 2 Department of Radiology, ARNAS G. Brotzu—Businco Oncologic Hospital, 09121 Cagliari, Italy; nicolas.papalexis@aob.it; 3 Diagnostic and Interventional Radiology Unit, IRCCS Istituto Ortopedico Rizzoli, 40136 Bologna, Italy; giancarlo.facchini@ior.it (G.F.); marco.miceli@ior.it (M.M.)

**Keywords:** spine, sacrum, sacral tumors, MRI, tumor, osteolysis, bone tumor, benign, malignant

## Abstract

The sacrum is a common site for a wide range of pathological processes, including benign and malignant tumors as well as non-neoplastic conditions. Accurate diagnosis of sacral lesions remains challenging due to overlapping imaging features and the anatomical complexity of the region. This review provides a comprehensive overview of sacral lesions, with emphasis on imaging characteristics, diagnostic challenges, and clinical implications. Key imaging findings are highlighted to help narrow the differential diagnosis. While certain imaging patterns may suggest specific etiologies, image-guided biopsy is often necessary for definitive diagnosis. Familiarity with the spectrum of sacral pathologies is essential for radiologists to effectively contribute to diagnosis, patient management, and multidisciplinary care.

## Simple Summary

The sacrum is a common site for a wide range of pathological processes, including benign and malignant tumors as well as non-neoplastic conditions. This comprehensive review provides an updated and structured overview of the full spectrum of sacral lesions, with emphasis on imaging features and diagnostic pitfalls. Our review also integrates a systematic diagnostic approach designed to support accurate characterization and multidisciplinary management of these complex lesions.

## 1. Introduction

The sacrum may be affected by a broad spectrum of pathological entities, ranging from benign to highly aggressive malignant neoplasms, as well as non-neoplastic conditions. Although sacral lesions are relatively uncommon compared to those occurring in other parts of the skeleton, their diagnosis remains challenging due to overlapping imaging characteristics and the deep, anatomically complex location of the sacrum. Tumors in this region may arise from osseous, cartilaginous, neural, or marrow elements, each exhibiting distinct imaging patterns that correlate with their histopathologic composition.

Accurate characterization of sacral lesions is crucial for appropriate clinical management and prognostic assessment. Cross-sectional imaging, particularly CT and MRI, plays a central role in lesion detection, delineation of local extent, and tissue characterization, whereas histopathologic confirmation is often required for a definitive diagnosis. Radiologists must therefore be familiar with the key imaging features that help narrow the differential diagnosis and guide biopsy planning or surgical intervention.

This article provides a comprehensive review of sacral lesions, illustrating their characteristic imaging appearances and emphasizing distinguishing features among benign, malignant, and non-neoplastic conditions. The goal is to promote a systematic, pattern-based approach to the evaluation of sacral masses, supporting accurate diagnosis and optimized patient care.

## 2. Giant Cell Tumor of Bone

Giant cell tumor of bone (GCTB) is an intermediate (locally aggressive) primary bone tumor of the sacrum, accounting for approximately 2–5% of all primary sacral tumors. It most often occurs in young adults (third to fourth decades). There is no marked sex predilection, although a slight female predominance has been reported in some series. Although it lacks overt malignant histologic features, GCTB can be locally destructive and is associated with a substantial rate of local recurrence following treatment [1,2,3]. In the axial skeleton, the sacrum is the most common location for GCTB, and the vertebral body is more frequently involved than the posterior elements.

Sacral GCTBs typically exhibit an eccentric location and may abut or extend across the sacroiliac joint. They usually appear as expansile, osteolytic lesions that remodel the bone and frequently breach the cortex to invade adjacent soft tissues. On computed tomography (CT), they manifest as purely lytic and destructive lesions without evidence of matrix mineralization or dystrophic calcifications. The margins are typically non-sclerotic, consistent with the infiltrative growth pattern [4].

On magnetic resonance imaging (MRI), GCTBs often display heterogeneous signal intensity, with a predominance of low-to-intermediate T2 signal due to hemosiderin deposition and high cellularity. Foci of intrinsic T1 hyperintensity related to intralesional hemorrhage are common, as are fluid–fluid levels resulting from secondary aneurysmal bone cyst changes. Bone marrow edema and soft-tissue extension are frequent findings, and post-contrast sequences typically show heterogeneous enhancement of the solid components [5,6].

**Figure 1:**
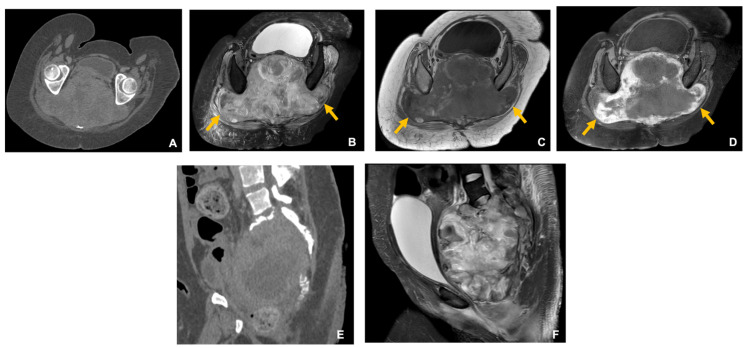
Giant cell tumor of bone in a 33-year-old woman. Axial (**A**) and sagittal (**E**) CT images show a very large osteolytic sacral lesion with cortical destruction. MRI sequences—axial (**B**) and sagittal (**F**) T2-weighted fat-suppressed, axial T1-weighted (**C**), and axial post-contrast fat-suppressed T1-weighted (**D**)—demonstrate intralesional T1 hyperintensity consistent with hemorrhage, low-to-intermediate T2 signal compatible with hemosiderin deposition, and heterogeneous enhancement. The lesion obliterates the expected pathways of the sciatic nerves (arrows).

In a young adult (20–40 years), the presence of a purely lytic, expansile lesion without calcifications, showing low-to-intermediate T2 signal intensity and hemorrhagic foci, strongly favors the diagnosis of giant cell tumor of bone over chordoma or chondrosarcoma (Figure 1).

## 3. Nerve Sheath Tumors

Nerve sheath tumors, including schwannomas and neurofibromas, account for approximately 8% of primary sacral tumors. They typically occur in young to middle-aged adults, between the second and fourth decades of life, with no clear sex predilection. These lesions most commonly present as intradural extramedullary masses, arising from neurogenic elements within the sacral canal, and may extend through the neural foramina into the presacral space [7,8].

On computed tomography (CT), nerve sheath tumors often cause smooth remodeling and widening of the sacral neural foramina, reflecting their slow, expansile growth. The lesions may appear as well-circumscribed, low-attenuation masses, occasionally containing areas of cystic degeneration [9].

On magnetic resonance imaging (MRI), these tumors typically exhibit high signal intensity on T2-weighted images and enhancement of the solid components following contrast administration. A “target sign”, characterized by a central T2 hypointense focus surrounded by a hyperintense peripheral rim, may be seen, particularly in neurofibromas, reflecting central fibrocollagenous tissue and peripheral myxoid change. Large schwannomas may demonstrate the classic dumbbell configuration, extending both within and beyond the sacral canal through the neural foramina, sometimes causing bone remodeling or erosion [9].

**Figure 2:**
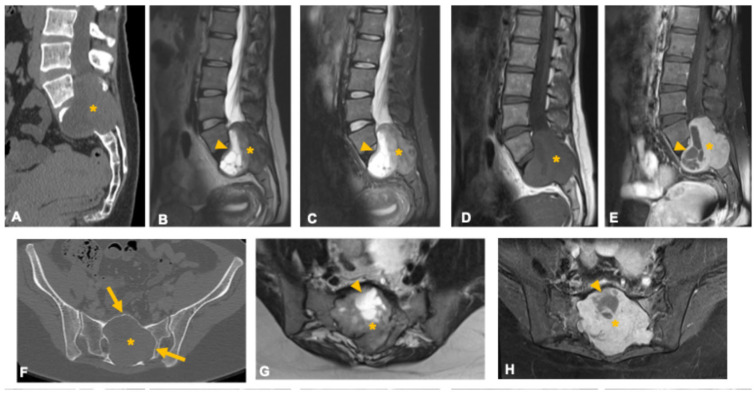
Giant invasive sacral schwannoma in a 34-year-old woman. Axial (**A**) and sagittal (**F**) CT images show a large sacral mass (asterisk) causing bone erosion and obliteration of the vertebral canal. The presence of sclerotic margins and smooth bone remodeling (arrows in (**F**)) suggests slow tumor growth. MRI sequences—T2-weighted (**B**,**G**), STIR (**C**), and T1-weighted (**D**) demonstrate a predominantly solid, heterogeneous mass (asterisks) with internal cystic areas (arrowheads) and clear delineation of canal and foraminal involvement. Post-contrast images (**E**,**H**) reveal intense enhancement of the solid components with non-enhancing cystic regions. Bone remodeling, extensive foraminal and canal involvement, and the presence of cystic areas support this diagnosis.

The presence of a well-defined, T2-hyperintense lesion causing foraminal expansion and showing the target sign or dumbbell morphology strongly supports the diagnosis of a benign peripheral nerve sheath tumor (Figure 2). Although the vast majority of sacral nerve sheath tumors are benign, malignant peripheral nerve sheath tumor (MPNST) should be considered in the differential diagnosis, particularly in patients with neurofibromatosis type 1 or when imaging shows worrisome features such as rapid growth, size > 5 cm, ill-defined margins, heterogeneous signal with necrosis or hemorrhage, perilesional edema, and infiltrative behavior or frank bone destruction.

## 4. Osteoid Osteoma

Osteoid osteoma is a benign but characteristically painful bone-forming tumor that typically affects males between 10 and 20 years of age. Spinal involvement occurs in fewer than 10% of cases, and only about 2% arise in the sacrum [10]. In the spine, osteoid osteoma involves the posterior vertebral elements. Approximately 75% of spinal osteoid osteomas are found in the posterior elements, which include the lamina, pedicles, articular facets, and spinous or transverse processes.

On computed tomography (CT), osteoid osteoma classically presents as a small, round, low-attenuation lesion with a central mineralized nidus measuring less than 2 cm, surrounded by an osteoblastic reaction that produces a rim of peripheral sclerosis. Magnetic resonance imaging (MRI) findings depend on the degree of mineralization: the nidus is usually hypointense on T1-weighted images and iso- to hypointense on T2-weighted sequences. Bone marrow edema and adjacent soft-tissue inflammatory changes are frequently imaging findings associated with osteoid osteoma [11].

**Figure 3:**
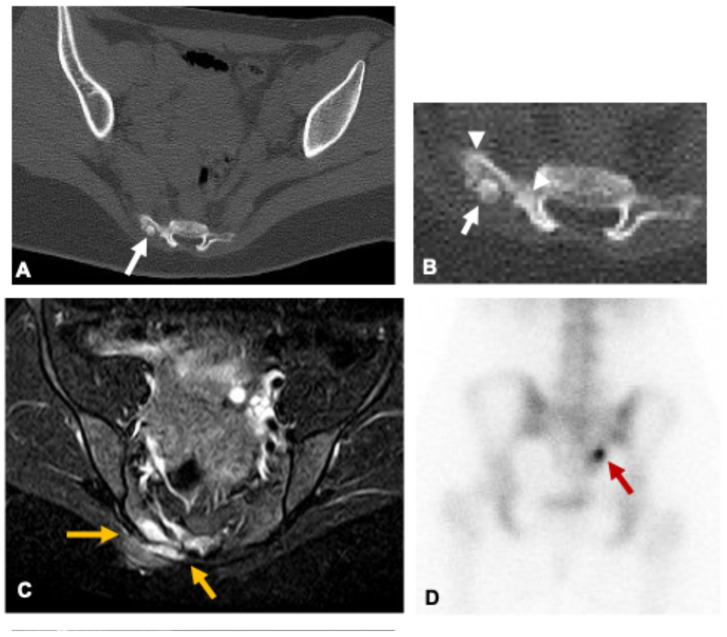
Osteoid osteoma in a 16-year-old girl. Axial CT images (**A**,**B**) show a small osteolytic lesion in the right lateral aspect of S3 with a central calcified nidus (white arrows) and surrounding sclerosis (arrowheads), findings typical of mineralized osteoid osteoma. On axial fat-saturated T2-weighted MRI (**C**), the nidus is not clearly visualized; instead, prominent bone marrow and adjacent soft-tissue edema are noted (yellow arrows). Bone scintigraphy (**D**) demonstrates focal radiotracer uptake (red arrow) consistent with high osteoblastic activity. The patient presented with persistent nocturnal sacral pain relieved by NSAIDs, associated with localized tenderness and occasional radiation to the right gluteal region.

In a young patient (10–25 years) presenting with localized pain and a small osteolytic lesion with or without central calcification associated with surrounding sclerosis and perilesional edema, osteoid osteoma should be strongly considered. Thin-slice CT is the most sensitive imaging modality for detecting the nidus and should always be included in the evaluation (Figure 3).

## 5. Osteoblastoma

Osteoblastoma is a benign but locally aggressive bone-forming neoplasm that shares histological features with osteoid osteoma but is distinguished by its larger size (typically exceeding 2 cm) and more expansive growth pattern. It is rare in the sacrum and occurs predominantly in males, most often during the second decade of life [10,11]. Like osteoid osteomas, when osteoblastomas affect the spine, they show a predilection for the posterior vertebral elements.

On computed tomography (CT), osteoblastoma typically appears as an expansile, lytic lesion with a well-defined sclerotic rim and internal calcifications of variable density. Although histologically benign, it may exhibit locally aggressive behavior, including cortical thinning or destruction and extension into adjacent soft tissues.

**Figure 4:**
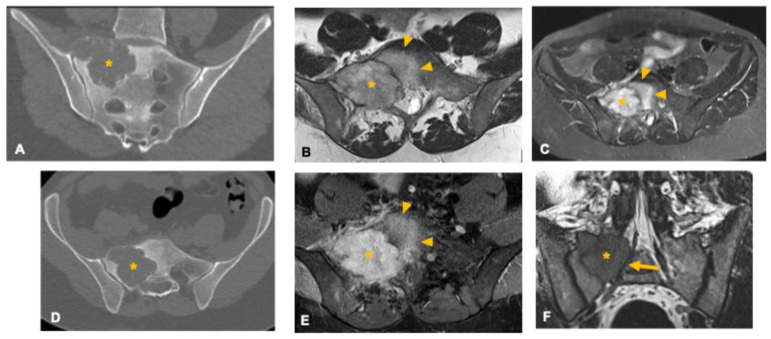
Osteoblastoma in a 33-year-old man. Coronal (**A**) and axial (**D**) CT images show a well-defined osteolytic lesion in the right sacral ala (asterisk) with lobulated contours, small internal calcifications, and surrounding sclerosis. The lesion demonstrates an expansile growth pattern with smooth scalloping of the anterior and posterior cortices, suggestive of slow progression. Axial T2-weighted (**B**), STIR (**C**), and post-contrast T1-weighted fat-suppressed (**E**) MR images depict an intermediate-to-high T2 signal lesion (asterisk) with diffuse enhancement and extensive perilesional edema (arrowheads), a hallmark imaging feature of osteoblastoma. Coronal volumetric T2-weighted image (**F**) demonstrates foraminal narrowing and radicular compression (arrow) caused by the expansile component.

Magnetic resonance imaging (MRI) findings may be nonspecific, as the lesion usually demonstrates intermediate signal intensity on both T1- and T2-weighted sequences. A hallmark feature is the presence of extensive peritumoral bone marrow and soft-tissue edema, which may be disproportionate to the lesion size [12] (Figure 4).

## 6. Aneurysmal Bone Cyst (ABC)

Aneurysmal bone cyst (ABC) is a benign, expansile, blood-filled lesion that typically affects young individuals (<30 years), with a slight female predominance and peak incidence during the second decade of life. It is relatively uncommon in the sacrum. In the spine, ABCs most commonly involve the posterior vertebral elements, although they may extend into the vertebral body. Histologically, ABCs consist of multiloculated cystic spaces filled with blood, separated by fibrous septa containing osteoclast-like giant cells and reactive bone formation. They may occur as primary lesions or, more frequently, secondary to other bone tumors, such as giant cell tumors of bone [13].

On computed tomography (CT), ABCs appear as well-defined, osteolytic, and expansile lesions with variable degrees of expansion and a thin peripheral rim of sclerosis. Magnetic resonance imaging (MRI) characteristically demonstrates multiple cystic cavities separated by internal septations, often showing fluid–fluid levels that reflect blood sedimentation of different ages. After contrast administration, there is enhancement of the lesion’s periphery and internal septa. These imaging findings, particularly the presence of fluid–fluid levels combined with septal enhancement, are highly suggestive of the diagnosis [14].

**Figure 5:**
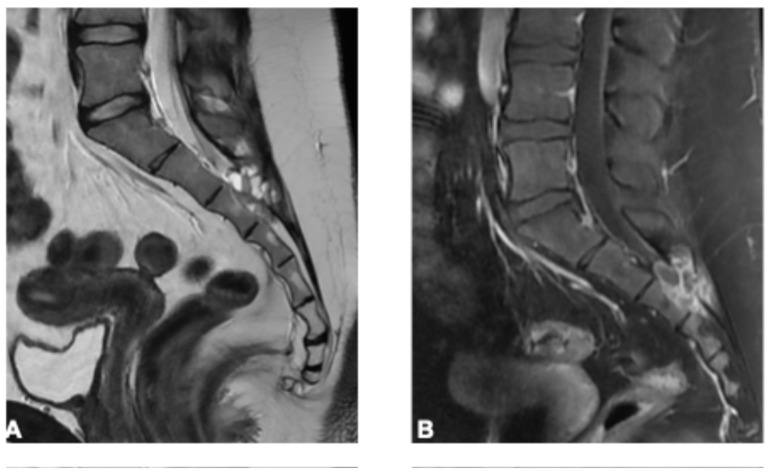
Aneurysmal bone cyst in a 12-year-old girl. Sagittal T2-weighted MRI (**A**) shows an expansile, multiloculated cystic lesion involving the posterior elements of the sacrum and extending into the spinal canal. The lesion demonstrates multiple fluid–fluid levels, consistent with hemorrhagic content. Post-contrast fat-suppressed T1-weighted image (**B**) reveals peripheral and septal enhancement.

In a young patient (10–30 years) with a posteriorly located sacral lesion exhibiting fluid–fluid levels, hemorrhagic content, and prominent perilesional edema, aneurysmal bone cyst should be the leading consideration, if no solid component is observed in post-contrast images (Figure 5). When a solid component is identified, a secondary ABC must be suspected, most commonly due to an underlying giant cell tumor of bone, or less frequently, telangiectatic osteosarcoma. However, many other bone lesions may eventually cause secondary ABC.

## 7. Intraosseous Hemangioma

Intraosseous hemangioma is a common benign vascular lesion of the spine and may involve the sacrum [9,11,12]. Most lesions are incidental. On CT, they typically demonstrate coarse vertically oriented trabeculae, producing a ‘polka-dot’ appearance on axial images and a ‘corduroy’ pattern on sagittal/coronal reconstructions [11,12]. Aggressive variants are uncommon but may present with cortical breach, extraosseous extension, or neurologic symptoms [9,11,12].

**Figure 6:**
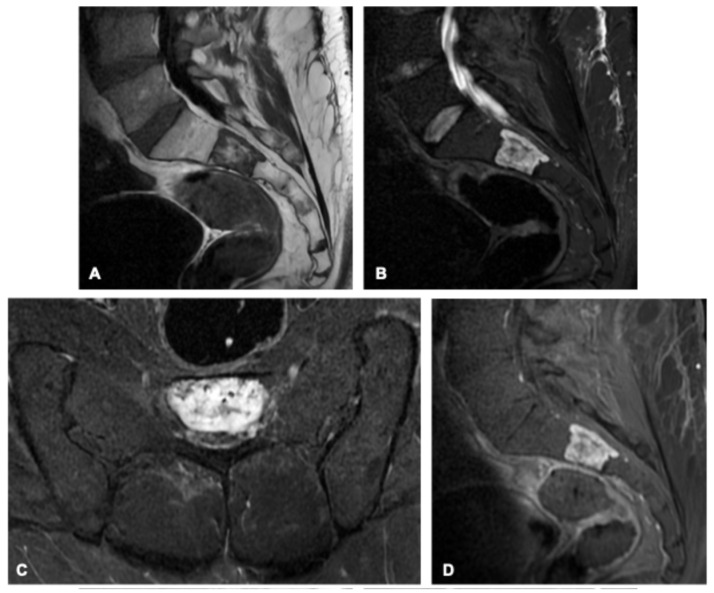
Intraosseous Hemangioma in a 44-year-old man. (**A**) Sagittal T1-weighted image shows a well-defined, predominantly T1-hyperintense intraosseous lesion in the S2 vertebral body, consistent with fatty stroma. (**B**) Sagittal T2 fat-suppressed image demonstrates marked hyperintensity. (**C**) Axial T2 fat-suppressed image best depicts thickened internal trabeculae, producing the characteristic punctate (“polka-dot”) pattern. (**D**) Post-contrast sagittal T1 fat-suppressed image shows enhancement of the lesion, without an extraosseous soft-tissue component.

On MRI, typical hemangiomas are hyperintense on both T1- and T2-weighted images due to fatty marrow content, with low-signal thickened trabeculae and variable enhancement [11,12] (Figure 6). Atypical hemangiomas may show lower T1 signal and more prominent enhancement, potentially mimicking metastasis; correlation with CT trabecular architecture and fat-sensitive sequences is helpful [11,12].

## 8. Chordoma

Chordoma is the most common primary malignant tumor of the sacrum, accounting for approximately 45% of all malignant sacral neoplasms. It predominantly affects males in the fifth decade of life and is characterized by locally aggressive behavior and a high rate of local recurrence [15,16,17].

Chordomas originate from intraosseous remnants of the embryonic notochord and typically arise as midline lesions involving the lower sacral segments, most often between S3 and S5. On computed tomography (CT), they appear as lobulated, expansile, osteolytic masses that frequently contain internal calcifications (in approximately 50–70% of cases) [18,19,20].

On magnetic resonance imaging (MRI), chordomas exhibit heterogeneously high signal intensity on T2-weighted images owing to their abundant myxoid stroma, interspersed with hypointense fibrous septa that impart a distinctive lobulated appearance. Areas of T2 hypointensity may also correspond to hemosiderin deposition from prior hemorrhage. On T1-weighted sequences, the tumor is generally hypointense to isointense, although focal hyperintense areas may be observed due to intratumoral hemorrhage or dense mucoid material. Following contrast administration, moderate to intense enhancement of the solid components is typically seen [21].

**Figure 7:**
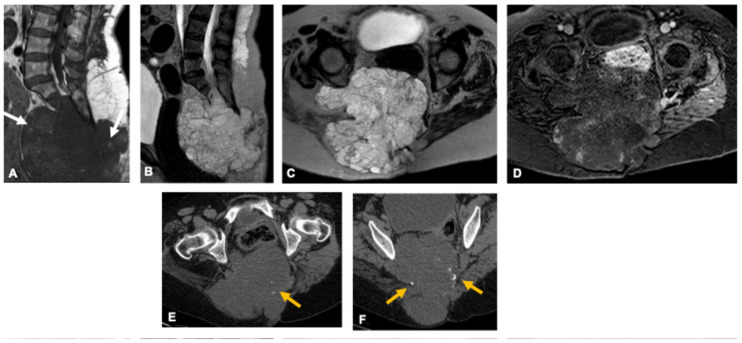
Chordoma in a 62-year-old woman. Sagittal T2-weighted MR image (**A**) and sagittal T1-weighted image (**B**) show an extensive osteolytic lesion centered in the sacrococcygeal region with cortical destruction and a large lobulated extraosseous component extending anteriorly into the pelvis and posteriorly into the spinal canal. The lesion exhibits high T2 signal intensity with internal hypointense septations and heterogeneous post-contrast enhancement (**D**). On T1-weighted imaging, focal areas of high signal (arrows in (**A**)) correspond to intratumoral hemorrhage or proteinaceous material. CT images (**C**,**E**,**F**) demonstrate an expansile destructive mass with amorphous peripheral calcifications (yellow arrows), representing residual bone sequestra rather than dystrophic calcification.

The combination of a midline, lower sacral location, destructive growth pattern, high T2 signal intensity with internal septations, and bone erosion is highly suggestive of chordoma. Peripheral calcifications and the presence of focal T1 hyperintensity further favor chordoma over chondrosarcoma, which more often demonstrates an eccentric location and ring-and-arc chondroid calcifications (Figure 7).

## 9. Chondrosarcoma

Chondrosarcoma is the second most common primary malignant tumor of the sacrum, following chordoma. It occurs more frequently in males, typically between the fourth and fifth decades of life, and tends to involve the upper sacral segments. Unlike chordomas, chondrosarcomas usually present as eccentric lesions with a lobulated, expansile morphology [22,23].

On computed tomography (CT), these tumors appear as large, lytic, and expansile masses containing chondroid matrix calcifications, which display the characteristic ring-and-arc pattern that reflects endochondral ossification within cartilaginous lobules [24].

On magnetic resonance imaging (MRI), calcified areas correspond to signal voids on all pulse sequences, whereas non-calcified cartilaginous regions are hyperintense on T2-weighted images and hypo- to isointense relative to skeletal muscle on T1-weighted sequences. After contrast administration, peripheral and septal (ring-and-arc) enhancement is typically observed in well-differentiated tumors (specially low grade chondrosarcoma), corresponding to the vascular septa between the cartilaginous lobules [25,26].

**Figure 8:**
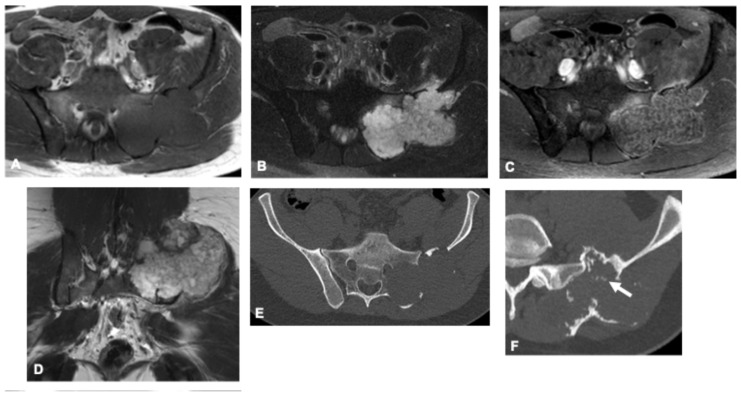
Chondrosarcoma in a 31-year-old man. Axial and coronal MR images (**A**–**D**) show an extensive osteolytic lesion involving the left sacral ala and iliac bone, demonstrating intermediate signal intensity on the T1-weighted image (**A**) and high signal on T2-weighted images (**B**,**D**) with thin hypointense internal septations and a characteristic lobulated contour. Post-contrast imaging reveals peripheral and septal enhancement (**C**), consistent with the cartilaginous nature of the tumor. Corresponding CT images (**E**,**F**) highlight the relative paucity of matrix mineralization, most pronounced in the cranial portion (arrows), typical of chondral-type matrix calcifications.

The presence of a chondroid matrix with ring-and-arc calcifications, lobulated architecture, and expansile but eccentric growth is characteristic of sacral chondrosarcoma. Involvement of the upper sacrum, an eccentric location, greater signal homogeneity on T1-weighted imaging, and the distinct pattern of chondroid calcifications help distinguish chondrosarcoma from chordoma (Figure 8).

## 10. Osteosarcoma

Osteosarcoma accounts for approximately 9% of all primary malignant tumors of the sacrum. It occurs more frequently in males, typically during the fourth decade of life, and tends to exhibit an eccentric location within the sacrum. Some cases arise secondarily, most often due to malignant degeneration of Paget’s disease, particularly in older adults [26].

On computed tomography (CT), osteosarcoma typically manifests as a predominantly osteoblastic and permeative lesion with cortical destruction and extension into adjacent soft tissues. CT is particularly valuable for identifying the osteoid matrix mineralization, which represents the hallmark of osteosarcoma and may appear as dense, amorphous areas of ossification [27].

On magnetic resonance imaging (MRI), the nonmineralized components of the tumor show nonspecific signal characteristics, appearing hypointense on T1-weighted and hyperintense on T2-weighted images, with heterogeneous post-contrast enhancement. The ossified portions of the lesion are markedly hypointense, exhibiting signal intensity similar to the cortical bone on all sequences [28].

**Figure 9:**
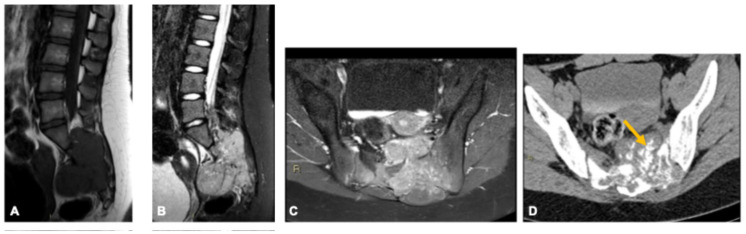
Osteosarcoma in a 9-year-old girl. Sagittal and axial MR images (**A**–**C**) demonstrate a large, eccentrically located lesion involving the left sacrum with cortical destruction, heterogeneous signal intensity, and extensive extraosseous soft-tissue extension. Post-contrast enhancement (**C**) is irregular, reflecting the tumor’s heterogeneous composition. Corresponding CT image (**D**) reveals mixed lytic and sclerotic changes with a dense, amorphous osteoid matrix (arrow).

The combination of aggressive bone destruction, osteoid matrix formation, and soft-tissue extension is highly suggestive of osteosarcoma. When present, a dense, amorphous osteoid matrix remains a distinctive imaging feature, aiding in differentiation from other malignant sacral neoplasms (Figure 9).

## 11. Lymphoma

Lymphoma involving the sacrum is extremely rare, accounting for approximately 1% of all non-Hodgkin lymphoma cases. It occurs more frequently in males, typically between the second and third decades of life. Sacral involvement may represent either primary osseous lymphoma or, more commonly, secondary infiltration from systemic disease [29].

On computed tomography (CT), lymphoma generally appears as a permeative, lytic lesion with minimal cortical destruction, often diffusely involving the sacrum and extending into adjacent soft tissues. Despite the degree of marrow infiltration, cortical integrity is usually better preserved compared with other aggressive malignancies [29].

On magnetic resonance imaging (MRI), the lesion demonstrates diffuse bone marrow replacement, with low signal intensity on T1-weighted images and high signal on T2-weighted sequences, typically accompanied by homogeneous post-contrast enhancement. The presence of regional or distant lymphadenopathy serves as an important diagnostic clue favoring lymphoma over other sacral neoplasms [30].

**Figure 10:**
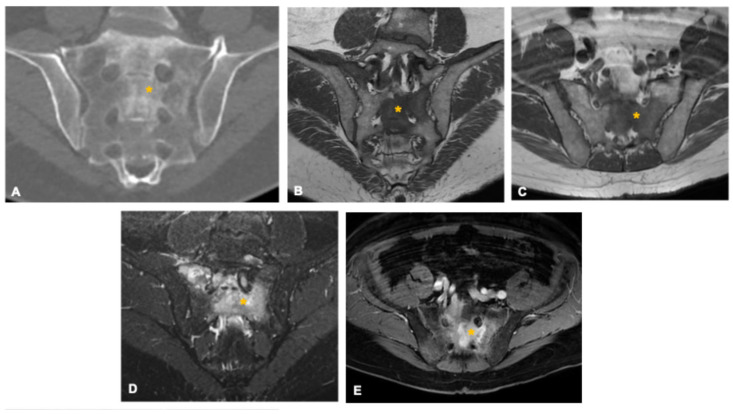
Primary bone lymphoma in a 71-year-old man. Coronal CT image (**A**) demonstrates a predominantly sclerotic, poorly defined sacral lesion (asterisk). Coronal (**B**) and axial (**C**) T1-weighted images, as well as coronal STIR (**D**), reveal a sacral lesion (asterisk) with low-to-intermediate signal intensity on T1 and predominantly high signal on T2, with poorly demarcated margins. On the post-contrast sequence (**E**) heterogeneous enhancement is observed, including a central focus of intense enhancement (asterisk), an important consideration when selecting a biopsy site to increase diagnostic yield, as inconclusive biopsy results are not uncommon in this type of neoplasm.

In cases presenting with an ill-defined mixed lesion (lytic and sclerotic) where percutaneous biopsy is inconclusive but malignancy remains suspected based on imaging findings, primary bone lymphoma should be strongly considered, and open biopsy may be warranted to establish a definitive diagnosis (Figure 10).

## 12. Ewing’s Sarcoma

Ewing’s sarcoma accounts for approximately 9% of all primary sacral tumors and typically affects males between 10 and 30 years of age. The sacral ala is the most common site of involvement [31].

On computed tomography (CT), it appears as an aggressive osteolytic lesion with cortical destruction and a prominent extraosseous soft-tissue mass. An aggressive periosteal reaction may be present, and diffuse sclerosis is observed in up to 70% of cases [32].

On magnetic resonance imaging (MRI), Ewing’s sarcoma typically demonstrates a T2-weighted hyperintense, infiltrative soft-tissue mass with heterogeneous enhancement after contrast administration, reflecting the coexistence of necrotic and viable tumor areas. Bone marrow replacement, periosteal reaction, and soft-tissue invasion are frequent findings [32,33].

**Figure 11:**
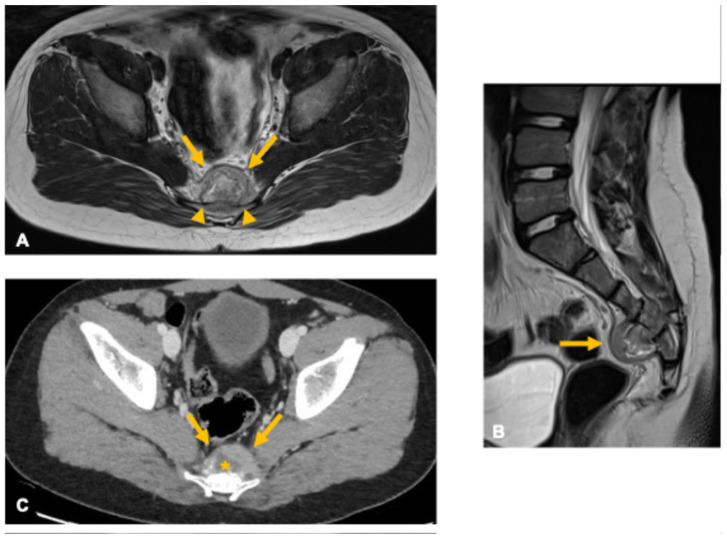
Ewing sarcoma in a 10-year-old boy. Axial and sagittal T2-weighted MR images (**A**,**B**) show a heterogeneous lesion centered in the lower sacrum (arrows) with a large anterior extraosseous soft-tissue component. Post-contrast CT image (**C**) demonstrates heterogeneous enhancement with central hypoenhancing areas suggestive of necrosis (asterisk). The lesion extends beyond the sacral cortex (arrowheads) with relatively preserved cortical margins, a characteristic feature of small round blue cell tumors such as Ewing sarcoma.

The combination of a destructive osteolytic lesion, prominent soft-tissue extension, and occurrence in a young patient (10–30 years) is highly characteristic of sacral Ewing’s sarcoma. The identification of a large extraosseous component with relative preservation of cortical margins further supports this diagnosis (Figure 11).

## 13. Ewing’s Multiple Myeloma and Plasmacytoma

Multiple myeloma and solitary plasmacytoma represent a spectrum of plasma cell neoplasms arising from the monoclonal proliferation of malignant plasma cells. Solitary plasmacytoma manifests as a single localized osseous lesion, whereas multiple myeloma is characterized by multifocal skeletal involvement. These entities occur more frequently in males over 60 years of age and predominantly involve the axial skeleton, including the spine, pelvis, and ribs [25,34].

In the sacrum, lesions are typically lytic, presenting as focal or diffuse bone destruction. On computed tomography (CT), they appear as multiple, sharply defined osteolytic defects with non-sclerotic margins, reflecting bone resorption without reactive sclerosis [35]. Magnetic resonance imaging is the most sensitive modality for detecting diffuse marrow involvement by myelomatous foci, which appear as nonspecific signal abnormalities [35].

**Figure 12:**
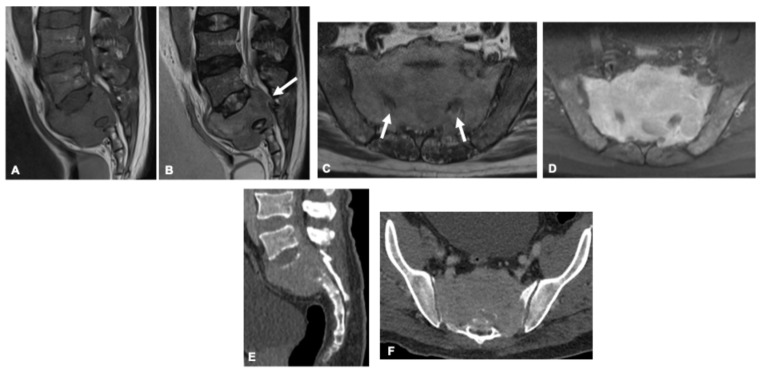
Plasmacytoma in a 61-year-old man. Sagittal and axial MR images (**A**–**D**) demonstrate a sacral osteolytic lesion with intermediate signal intensity on T1-weighted (**A**) and T2-weighted images (**B**,**C**), and diffuse, homogeneous post-contrast enhancement (**D**). Corresponding contrast-enhanced CT images (**E**,**F**) show extensive cortical destruction with extraosseous soft-tissue extension and marked narrowing of the spinal canal and sacral foramina, more evident on MRI (arrows).

The presence of multiple well-circumscribed osteolytic lesions without marginal bone reaction in an older adult, involving the axial skeleton and showing diffuse marrow replacement on MRI, strongly suggests multiple myeloma or plasmacytoma, depending on the extent of skeletal involvement (Figure 12).

## 14. Perivascular Epithelioid Cell Tumor (PEComa)

Perivascular epithelioid cell tumor (PEComa) is a rare mesenchymal neoplasm composed of perivascular epithelioid cells that display a distinctive perivascular growth pattern and variable degrees of myomelanocytic differentiation. It is extremely uncommon, with the sacrum representing an exceptional site of occurrence [36,37].

On computed tomography (CT), PEComa presents as a well-demarcated, osteolytic, and expansile lesion that may show cortical thinning or destruction and extension into adjacent soft tissues [38].

**Figure 13:**
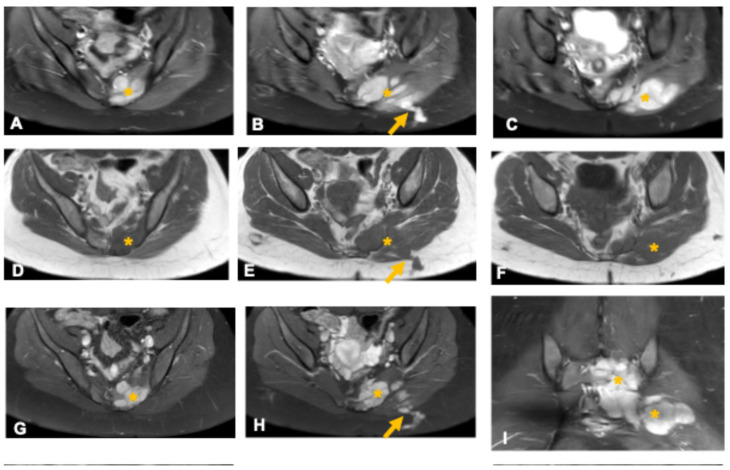
Sequential axial and coronal MR images (**A**–**I**) demonstrate an expansile lesion (asterisk) involving the lower left sacrum. The lesion shows predominantly high signal intensity on T2-weighted fat-suppressed images (**A**–**C**), low signal intensity on T1-weighted sequences (**D**–**F**), and diffuse heterogeneous enhancement on post-contrast fat-suppressed images (**G**–**I**). It has lobulated margins and extends extraosseously into the gluteal muscles and subcutaneous tissue, following the course of vascular structures, including perforating vessels (arrows). The elongated configuration and perivascular extension are suggestive imaging features of PEComa, although findings remain nonspecific.

On magnetic resonance imaging (MRI), the lesion generally appears iso- to hypointense relative to muscle on T1-weighted images and hyperintense on T2-weighted sequences, with heterogeneous enhancement following contrast administration [39] (Figure 13).

Although imaging features are nonspecific, the underlying perivascular arrangement of tumor cells, not directly appreciable radiologically, may correlate with marked internal vascularity and heterogeneous post-contrast enhancement, which can raise suspicion for this rare entity in the appropriate clinical and pathologic context.

## 15. Infection

Sacral osteomyelitis is a common complication of sacral pressure ulcers, especially those that have reached an advanced stage. More rarely, sacral osteomyelitis may be seen as an extension of infectious sacroiliitis. Infectious processes involving the sacrum, such as osteomyelitis, may closely mimic neoplastic lesions on imaging, especially when pressure ulcers and sacroiliitis are not present to help define the correct diagnosis. In the case of hematogenous osteomyelitis, the presence of multiple lesions, surrounding bone marrow edema, and adjacent soft-tissue inflammatory changes should raise suspicion for infection rather than tumor.

**Figure 14:**
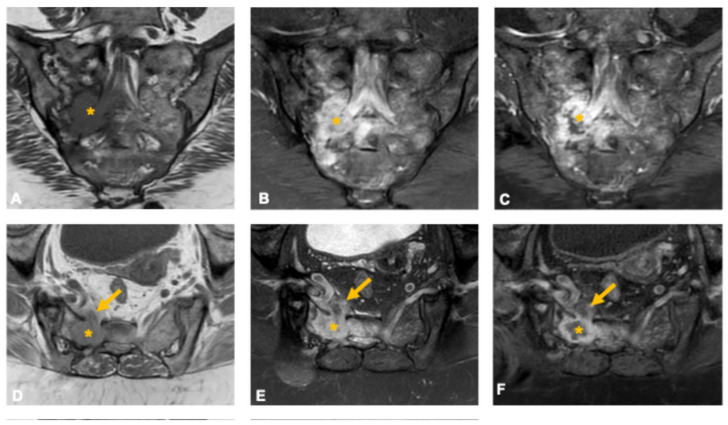
Paracoccidioidomycosis bone lesion in a 54-year-old woman. Coronal and axial MR images (**A**–**F**) show a destructive lesion centered in the right sacral ala (asterisk) with intermediate signal intensity on T1-weighted imaging (**A**,**D**), intermediate-to-high signal on T2-weighted Imaging (**B**,**E**) and heterogeneous enhancement with a central hypoenhancing area (**C**,**F**). Perilesional bone edema is noted, with extension of the edema/inflammatory process into the right sacral foramen, involving the neural root (arrows).

On computed tomography, acute infections typically appear as osteolytic, mass-like lesions with poorly defined margins and associated periarticular inflammatory fat stranding. Magnetic resonance imaging findings are often nonspecific, demonstrating areas of low signal intensity on T1-weighted and high signal intensity on T2-weighted sequences due to bone marrow edema and inflammatory infiltration. Following contrast administration, irregular and heterogeneous enhancement of both bone and adjacent soft tissues is commonly observed, which can help differentiate infection from neoplastic processes [9,40,41] (Figure 14).

Chronic osteomyelitis, commonly seen in sacral pressure ulcers, usually manifests with bone sclerosis, and in some cases, there may also be bone loss related to previous surgical debridement.

## 16. Metastases

Metastases represent the most common malignant neoplasms involving the sacrum, typically affecting patients over 40 years of age. The most frequent primary sources include carcinomas of the lung, breast, prostate, kidney, and gastrointestinal tract, as well as head and neck malignancies and melanoma. The presence of a known primary tumor and multiple skeletal lesions should immediately raise concern for metastatic disease [42].

On computed tomography (CT), sacral metastases most commonly appear as osteolytic lesions with cortical destruction and variable soft-tissue extension. However, prostate and breast carcinoma metastases often display a sclerotic or osteoblastic pattern. Less frequently, mixed lytic–sclerotic lesions may be encountered [42].

**Figure 15:**
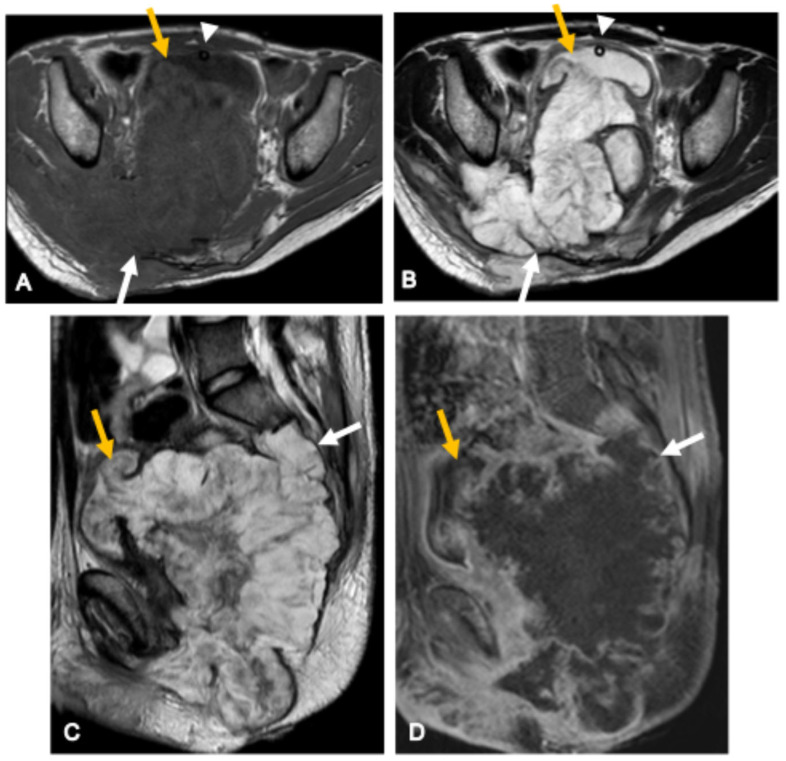
Intestinal adenocarcinoma with sacral invasion in a 50-year-old man. Axial T1-weighted (**A**) and T2-weighted (**B**) images, as well as sagittal T2-weighted (**C**) and contrast-enhanced fat-suppressed T1-weighted (**D**) images, demonstrate a large expansile pelvic mass invading the sacrum (white arrows) and urinary bladder (yellow arrows). The lesion exhibits predominantly high T2 signal intensity, peripheral enhancement after contrast administration, and extensive central necrosis. A Foley catheter is visible within the bladder (arrowheads).

Magnetic resonance imaging (MRI) provides superior sensitivity for detecting early marrow infiltration, with most lesions demonstrating low signal intensity on T1-weighted images and high signal intensity on T2-weighted or STIR sequences. Metastases from hypervascular primaries, such as renal cell carcinoma or thyroid carcinoma, may appear expansile and show prominent post-contrast enhancement (Figure 15).

The imaging appearance of sacral metastases is highly variable, reflecting the biological behavior and matrix composition of the underlying primary malignancy. Accurate recognition of these patterns, combined with clinical and oncologic correlation, is essential for appropriate diagnosis and management.

## 17. Osteoradionecrosis

Osteoradionecrosis is a form of radiation-induced ischemic necrosis that affects bone and adjacent soft tissues in the absence of residual, recurrent, or metastatic tumor. It results from vascular compromise, osteocyte loss, and impaired bone remodeling secondary to radiotherapy [43].

Although spinal involvement is uncommon, osteoradionecrosis may occur in patients who have received high-dose radiation to the pelvis or lower spine. On computed tomography (CT), it typically appears as an osteolytic lesion with irregular surrounding sclerosis, often mimicking metastatic disease. However, the presence of dense sclerotic bone, lack of periosteal reaction, and absence of a discrete soft-tissue mass help differentiate osteoradionecrosis from tumor recurrence [43].

On magnetic resonance imaging (MRI), affected regions demonstrate low signal intensity on T1-weighted images and variable T2 signal intensity, reflecting a mixture of necrotic bone, fibrosis, and marrow edema. Post-contrast enhancement is typically minimal or peripheral, depending on the degree of vascular compromise [43].

**Figure 16:**
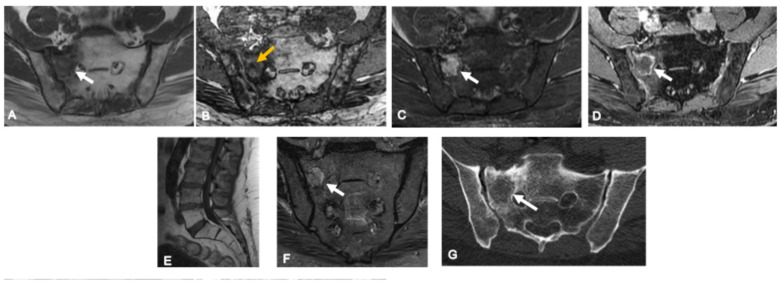
Osteoradionecrosis in a 56-year-old man. Follow-up imaging after radiotherapy for colorectal cancer demonstrates band-like fatty marrow replacement in the lumbosacral spine, consistent with post-radiation changes. A focal lesion is seen in the right sacral ala (white arrows), showing central rarefaction with peripheral sclerosis on CT (**G**), intermediate signal intensity on T1-weighted images (**A**,**E**), intermediate-to-high signal on STIR (**C**,**F**), and peripheral enhancement after contrast administration (**D**). The out-of-phase image (**B**) reveals focal signal drop-out (yellow arrow), favoring the presence of intralesional fat and making active metastatic disease less likely. CT-guided biopsy was performed to exclude metastasis, and histopathology confirmed osteonecrosis without evidence of malignancy.

When evaluating the sacrum after radiotherapy, both osteoradionecrosis and insufficiency fractures should be considered. The presence of peripheral enhancement and preserved fatty marrow signal within the lesion favors the diagnosis of osteoradionecrosis (Figure 16). Recognition of these characteristic imaging features in the appropriate clinical context is essential to avoid misdiagnosis and prevent unnecessary biopsy or intervention.

## 18. Bone Marrow Necrosis

Bone marrow necrosis is a rare condition characterized by ischemic necrosis of the hematopoietic and stromal components of the bone marrow, while the trabecular bone architecture remains relatively preserved. It is distinct from both avascular (osteonecrosis) necrosis and marrow aplasia and is most commonly associated with hematologic malignancies, though it may also occur secondary to severe infection, sepsis, sickle cell disease [44] or chemotherapeutic toxicity [45].

On computed tomography (CT), findings are often subtle, typically manifesting as ill-defined osteolytic lesions with variable sclerosis, which may be absent in early stages. Magnetic resonance imaging (MRI) is more sensitive, usually demonstrating a geographic pattern of marrow signal alteration, characterized by central areas of T1 and T2 hypointensity surrounded by a serpiginous T2-hyperintense rim. After contrast administration, peripheral rim enhancement is frequently observed, corresponding histologically to reactive granulation tissue [46,47].

**Figure 17:**
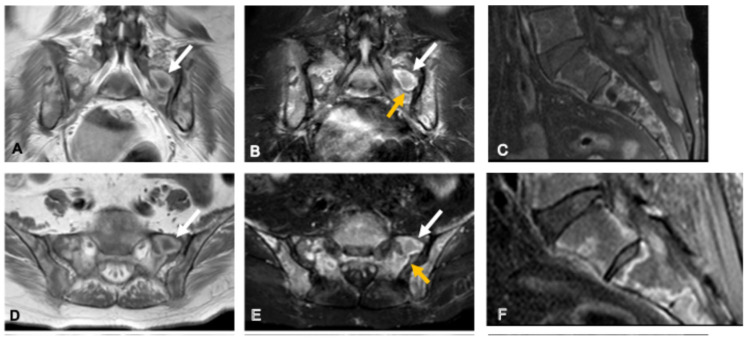
Bone marrow necrosis in a 56-year-old woman with acute lymphoblastic leukemia. Coronal T1-weighted (**A**) and fat-suppressed T2-weighted (**B**) images, sagittal contrast-enhanced fat-suppressed T1-weighted (**C**), axial T1-weighted (**D**), fat-suppressed T2-weighted (**E**), and sagittal contrast-enhanced fat-suppressed T1-weighted (**F**) sequences show extensive signal abnormalities involving the sacrum and iliac bones. The lesions exhibit areas of low signal intensity surrounded by a serpiginous rim of high signal intensity on both T1- and T2-weighted images (white arrows), producing a geographic pattern. Some lesions demonstrate an additional outer rim of low signal intensity on T2-weighted images (yellow arrows). Post-contrast images (**C**,**F**) reveal peripheral enhancement corresponding to reactive granulation tissue. The overall imaging appearance is atypical for lymphomatous infiltration and more consistent with bone infarction. CT-guided biopsy confirmed bone marrow necrosis, identified at leukemia diagnosis prior to treatment initiation.

**Figure 18:**
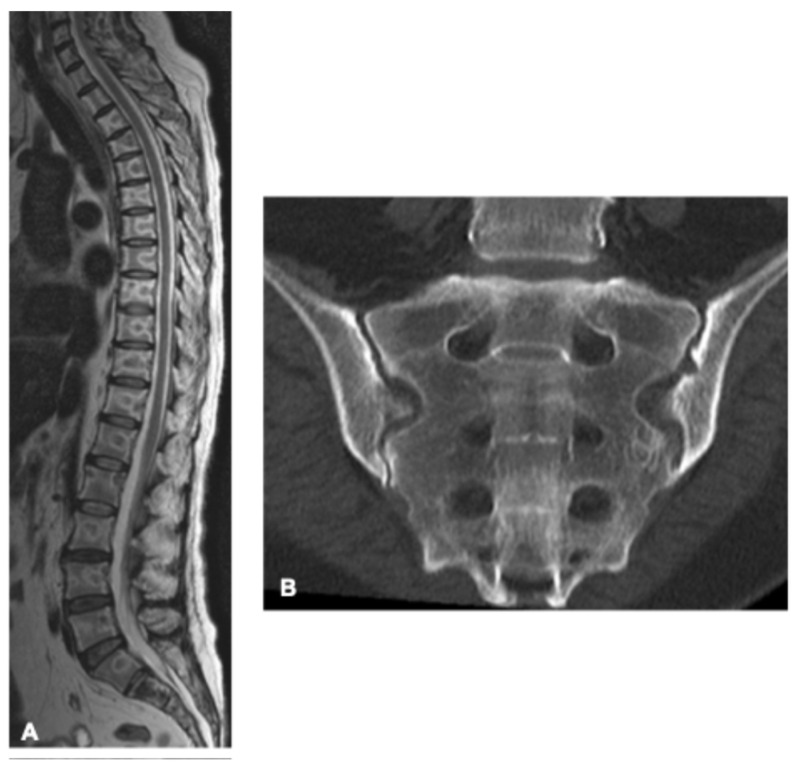
Bone marrow necrosis in a 56-year-old woman with acute lymphoblastic leukemia. Sagittal T2-weighted MRI of the thoracolumbar spine (**A**) demonstrates similar findings in multiple vertebral bodies. CT (**B**) obtained four days after MRI, performed to guide the bone biopsy, shows no significant changes, as trabecular bone architecture is preserved in this pathology, making CT less sensitive, particularly in the early stages.

This combination of imaging findings, particularly the serpiginous hyperintense border on T2-weighted images, peripheral enhancement, and preserved trabecular framework, is suggestive of bone marrow necrosis in the appropriate clinical context (Figure 17 and Figure 18). A more extensive and diffuse pattern of involvement, especially affecting the spine and pelvis, favors the diagnosis of bone marrow necrosis over osteonecrosis (avascular necrosis). When this diagnosis is suspected, evaluation for underlying hematologic malignancy is warranted due to its strong clinical association.

## 19. Sacral Insufficiency Fractures

Sacral insufficiency fractures are a subtype of stress fractures, being the result of normal stresses on abnormal bone, and fall under the broader group of pelvic insufficiency fractures. They are most frequently secondary to osteoporosis bone fragility, although many other conditions may be a risk factor, such as rheumatoid arthritis, Paget disease, osteomalacia, radiation therapy and pregnancy. Sacral insufficiency fractures are usually seen in elderly females who present with low back pain without any history of significant trauma. These fractures may be clinically confused with bone metastases, especially in cases of known prior malignancy, and imaging studies play a key role in the differential diagnosis [48].

Radiographs may initially be negative or show poorly defined areas of bone sclerosis. In more advanced cases, bone sclerosis may be better defined and vertically oriented in the sacral ala, and a radiolucent irregular fracture line may be identified within the sclerosis. CT has higher sensitivity than plain radiographs and may show a fracture line along with sclerosis that is parallel to the sacroiliac joint. CT imaging is less sensitive as compared to MRI and nuclear imaging [48].

**Figure 19:**
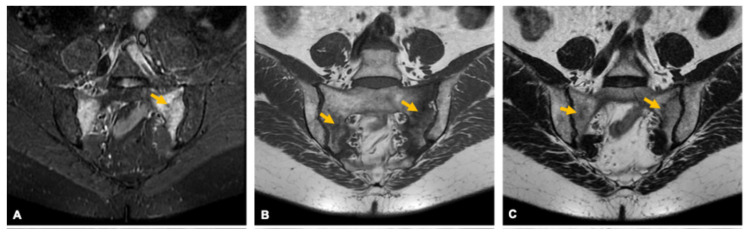
Sacral insufficiency fracture in a 68-year-old woman. Coronal MRI images in STIR (**A**), T1 (**B**), and T2 (**C**) sequences show fracture lines involving both sacral alae, oriented parallel to the sacroiliac joints (arrows), associated with marked bone marrow edema (**A**).

Nuclear medicine uptake of Tc-99m MDP is also sensitive but not specific. A typical H-sign or Honda sign (uptake in H pattern) may be present and is considered diagnostic. MRI is the most appropriate method for investigating sacral insufficiency fractures, as it has high sensitivity and specificity, and does not use ionizing radiation (Figure 19).

## 20. Systematic Approach to Sacral Lesions

Table 1 summarizes key demographic (age/sex), clinical, and imaging characteristics of the primary tumors, tumor-like lesions, and major mimics discussed in this review, including typical location within the sacrum, key CT/MRI findings, and main differential diagnoses/pitfalls, to facilitate side-by-side comparison.

Given the broad spectrum of primary and secondary entities that may involve the sacrum, a structured imaging approach is essential to narrow the differential diagnosis and guide appropriate management. Although overlapping imaging appearances are common, systematic evaluation of key morphologic and contextual parameters typically allows differentiation between benign and aggressive processes, and further distinction among specific tumor types.

The initial step consists of assessing lesion margins, cortical integrity, and enhancement pattern. Lesions with a well-defined sclerotic rim and preserved cortex are most consistent with benign pathology, whereas cortical thinning, destruction, or an ill-defined transition zone indicates an aggressive or malignant nature. Intense post-contrast enhancement and extension into adjacent soft tissues further support aggressiveness.

Subsequently, determining whether the lesion is solitary or multiple refines the diagnostic spectrum. Solitary aggressive lesions are most frequently represented by chordoma, chondrosarcoma, osteosarcoma, Ewing sarcoma, and giant cell tumor of bone (GCT), while multiple aggressive lesions more commonly correspond to metastases, lymphoma, or multiple myeloma. In contrast, benign solitary lesions encompass benign notochordal cell tumor (BNCT), osteoid osteoma, osteoblastoma, osteochondroma, cystic osteoid anomaly (COA), germ cell tumor, and neurogenic tumors. Multiple benign lesions typically include hemangiomas, enchondromatosis, or osteochondromatosis.

Further characterization relies on tumor-specific features:

- Matrix mineralization provides valuable diagnostic clues. “Rings and arcs” calcifications suggest a chondroid matrix, as seen in chondrosarcoma, whereas “cloud-like” osteoid mineralization is typical of osteosarcoma. A central nidus with variable mineralization and surrounding sclerosis is characteristic of osteoid osteoma and osteoblastoma.
- Lesion location within the sacrum also contributes to narrowing the differential: midline involvement favors chordoma or BNCT, while eccentric anterior or presacral masses are more characteristic of giant cell tumor of bone, osteosarcoma, or metastasis. Lesions centered in the posterior elements are typical of osteoid osteoma, osteoblastoma, or osteochondroma.
- MRI signal characteristics offer additional specificity. T1 hyperintensity reflects the presence of fat, blood, or proteinaceous material, commonly observed in hemangioma, BNCT, and GCT. T2 hyperintensity is a hallmark of chordoma, chondrosarcoma, and metastatic disease, whereas low T2 signal intensity may be seen in GCT, osteosarcoma, or osteoid osteoma due to fibrous or mineralized content.
- Patient age remains an important contextual determinant. Ewing sarcoma, osteoid osteoma, GCT, aneurysmal bone cyst (ABC), Langerhans cell histiocytosis (LCH), and germ cell tumors typically occur in patients younger than 30 years; chordoma, chondrosarcoma, and osteosarcoma are more common in middle-aged adults (30–60 years); and metastases or myeloma predominate in patients older than 60 years.

Cross-sectional imaging modalities are complementary. CT is superior for detecting cortical destruction, matrix mineralization, and subtle bone remodeling, while MRI provides excellent soft tissue contrast for evaluating neural, vascular, and visceral involvement. PET-CT, although limited in specificity, contributes to staging, assessment of multifocal disease, and evaluation of treatment response.

Attention should also be given to sacroiliac joint and pelvic organ involvement, which indicates local aggressiveness and has major surgical implications. Sacroiliac joint invasion is typical of GCT, primary osseous sarcoma, metastasis, and myeloma, and often necessitates extended resection and reconstruction to maintain pelvic stability.

Altogether, integrating morphological, topographical, and clinical parameters, border definition, cortical behavior, multiplicity, matrix composition, anatomic location, MRI signal characteristics, and patient demographics provides a reproducible and clinically meaningful framework for the diagnostic assessment of sacral lesions, facilitating accurate characterization and guiding therapeutic decision-making.

## 21. Conclusions

Sacral tumors encompass a wide spectrum of benign and malignant entities with overlapping clinical and imaging features. In this review, the diversity of sacral lesions was synthesized and discussed, with emphasis on epidemiologic aspects, clinical presentations, and the complementary roles of CT and MRI in lesion characterization. Applying a systematic approach that integrates lesion morphology, matrix composition, multiplicity, anatomical location, and patient demographics allows radiologists to narrow the differential diagnosis and guide appropriate management.

## Figures and Tables

**Table 1:** Summary of demographic and imaging features of sacral lesions (age/sex, typical location, key CT/MRI findings, and differential diagnoses/pitfalls).

Entity	Age/Sex	Predominant Location	CT Key Findings	MRI Key Findings	Key Differentiators/Pitfalls
**Benign Tumors**					
Giant cell tumor of bone (GCTB)	20–40 (F > M)	Eccentric; may abut/extend across SI joint	Purely lytic, expansile; non-sclerotic margin; no matrix mineralization; cortical breach/soft-tissue common	T1 low-iso with hemorrhagic foci (T1 high); T2 low-intermediate; fluid-fluid levels if secondary ABC; heterogeneous enhancement	Low-intermediate T2 + hemorrhage favors GCTB; secondary ABC may obscure solid component
Peripheral nerve sheath tumor (schwannoma/neurofibroma)	20–50 (no marked predilection)	Sacral canal/foramina; dumbbell extension	Well-circumscribed; smooth foraminal widening; remodeling; cystic degeneration possible	T1 low-iso; T2 high; target sign (esp. neurofibroma); solid enhancement with nonenhancing cystic areas	Smooth remodeling supports slow growth; large lesions can erode bone and mimic malignancy. Consider MPNST when there is rapid growth, large size, ill-defined margins, infiltrative behavior, marked heterogeneity with necrosis or hemorrhage, perilesional edema, or destructive bone change (especially in NF1).
Osteoid osteoma	10–25 (M > F)	Posterior elements; rare sacrum	Nidus < 2 cm (often calcified) + surrounding sclerosis; thin-slice CT best	Nidus T1 low; T2 variable; prominent marrow/soft-tissue edema; nidus enhances	MRI may miss nidus; edema can mimic infection or tumor
Osteoblastoma	10–30 (M > F)	Posterior elements; rare sacrum	Expansile lytic lesion > 2 cm; sclerotic rim; variable internal calcifications; cortex may be thinned or breached	T1/T2 intermediate; marked edema; diffuse enhancement	May appear locally aggressive; correlate with matrix and typical age/location
Aneurysmal bone cyst (ABC)	<30 (no marked predilection)	Posterior elements; expansile	Expansile lytic lesion; thin rim of sclerosis; septations	Multiloculated cystic lesion with fluid-fluid levels; peripheral/septal enhancement; no solid enhancing component in primary ABC	Solid enhancing component suggests secondary ABC (often GCTB) or telangiectatic osteosarcoma
Intraosseous hemangioma	40–60 (slight F > M)	Vertebral body; often incidental	Thickened trabeculae; ‘polka-dot’/‘corduroy’ pattern; usually non-aggressive	Typically T1 and T2 high (fat-rich) with low-signal trabeculae; enhancement variable; atypical lesions may be T1 low	Differentiate from metastasis, plasmacytoma, lymphoma; look for characteristic trabecular pattern and fat content; consider aggressive hemangioma if extraosseous extension
**Malignant tumors/hematologic malignancy**					
Chordoma	40–70 (M > F)	Midline, lower sacrum (S3–S5)	Lobulated destructive lytic mass; internal calcifications/bone sequestra; large soft-tissue component	T1 low-iso (focal T1 high if hemorrhage or protein); T2 very high with hypointense septa; moderate-intense enhancement	Midline lower sacrum + very high T2 typical; overlaps with chondrosarcoma
Chondrosarcoma	30–60 (M > F)	Eccentric; upper sacrum	Lytic expansile lesion with chondroid ring-and-arc calcifications	T1 low-iso; T2 high; peripheral/septal (ring-and-arc) enhancement; signal voids in calcified areas	Ring-and-arc calcification + eccentric location favor chondrosarcoma over chordoma
Osteosarcoma	30–50 (or older if Paget)	Often eccentric	Aggressive permeative/destructive lesion; osteoid matrix (dense amorphous mineralization); soft-tissue extension	Nonmineralized tumor T1 low, T2 high; mineralized portions markedly low on all sequences; heterogeneous enhancement	Osteoid matrix on CT is key; can mimic mixed metastasis
Lymphoma (primary/secondary)	Any age	Diffuse marrow involvement; may be multifocal	Permeative lytic lesion with relatively preserved cortex; soft-tissue mass possible	Diffuse marrow replacement (T1 low, T2 high); often homogeneous enhancement; lymphadenopathy is a clue	Cortex may appear preserved despite extensive disease; correlate with systemic findings
Ewing sarcoma	10–30	Often sacral ala	Aggressive lytic lesion; cortical destruction; large soft-tissue mass; periosteal reaction; sclerosis possible	T1 low; T2 high; heterogeneous enhancement; necrosis common	Age + large extraosseous component supports diagnosis; overlaps with osteomyelitis
Multiple myeloma/plasmacytoma	>60	Often multiple lesions in axial skeleton	Multiple well-defined lytic lesions; non-sclerotic margins; cortical destruction may be present	T1 low; T2/STIR high; enhancement variable (often homogeneous in plasmacytoma); diffuse marrow involvement on MRI	Multiplicity and clinical context key; solitary plasmacytoma may mimic metastasis
Metastases	>40	Variable; often multiple	Usually lytic; may be sclerotic (prostate/breast) or mixed; cortical destruction/soft-tissue variable	T1 low; T2/STIR high; enhancement variable; hypervascular primaries may show avid enhancement	Highly variable; known primary tumor and multiplicity are major clues
Perivascular epithelioid cell tumor (PEComa)	Variable (rare)	Variable	Well-demarcated lytic expansile lesion; cortical thinning/destruction; soft-tissue extension	T1 low-iso; T2 high; heterogeneous enhancement; may show prominent internal vascularity	Imaging nonspecific; diagnosis relies on pathology; consider in atypical lytic hypervascular lesion
**Non-neoplastic conditions/mimics**					
Infection (osteomyelitis)	Any	Often adjacent to ulcer/SI joint or hematogenous	Ill-defined lytic, mass-like lesion; inflammatory fat stranding; possible sclerosis/sequestrum in chronic cases	T1 low, T2 high (edema); irregular/heterogeneous enhancement; adjacent soft-tissue inflammation	Can closely mimic tumor; look for ulcer/sacroiliitis, systemic signs, and inflammatory soft tissues
Osteoradionecrosis	Post-radiotherapy	Within irradiated field	Osteolytic change with irregular surrounding sclerosis; typically no discrete soft-tissue mass	T1 low with variable T2; minimal or peripheral enhancement; preserved fatty marrow may help	Pitfall vs. recurrence/metastasis; correlate with radiation history and lack of mass
Bone marrow necrosis	Often hematologic malignancy/chemo/sepsis/sickle cell	Often multifocal (spine/pelvis)	Often subtle/normal early; preserved trabecular framework	Geographic marrow pattern with serpiginous rim; peripheral rim enhancement	CT can be insensitive; imaging may mimic metastasis/osteonecrosis; clinical context essential
Sacral insufficiency fracture	Elderly/osteoporotic (F > M)	Sacral alae, parallel to SI joints	Fracture line with vertical sclerosis; CT more sensitive than radiographs but less than MRI early	Marrow edema on STIR/T2; fracture line; often bilateral; Honda sign on scintigraphy may be present	Common mimic of metastasis; look for fracture line and classic distribution; correlate with risk factors

## Data Availability

The original contributions presented in this study are included in the article. Further inquiries can be directed to the corresponding author.
